# Evaluation of the Older Person Mental Health First Aid Course: Effects on Knowledge, Stigmatizing Attitudes, and Helping Behaviors

**DOI:** 10.1177/07334648251352309

**Published:** 2025-07-08

**Authors:** Julia Lyons, Kathy Bond, Betty Kitchener, Fairlie Cottrill, Anthony Jorm, Alyssia Rossetto, Claire Kelly, Nicola Reavley, Amy Morgan

**Affiliations:** 1 2281 The University of Melbourne, Melbourne, Australia; 2 Mental Health First Aid Australia, Melbourne, Australia

**Keywords:** mental health first aid, older people, early intervention, stigma, helping behavior, dementia, depression

## Abstract

The Older Person Mental Health First Aid® (MHFA®) course teaches adults practical skills to support older adults (65+ years) who are developing a mental health problem or crisis, including depression, anxiety, psychosis, substance use problems, confusion, and dementia. This study aimed to evaluate its effectiveness in a sample of Australian adults attending the course. Outcomes were assessed before the course, immediately after, and six months later. Measures assessed the participant’s knowledge, stigmatizing attitudes, willingness to help, confidence in- and quality of intended, and actual helping behavior toward an older person with depression or dementia. 148 Australian participants completed the pre-course survey, 133 at post-course and 67 at six-month follow-up. Significant improvements were observed at post and six-month follow-up on knowledge, desire for social distance, confidence, and quality of intended help toward dementia. The Older Person MHFA course may be an effective early intervention education course for supporting older people’s mental health.

**What this paper adds**

- This paper provides evidence for the effectiveness of an educational course on supporting older people who are developing a mental health problem or crisis.
- The evaluation improved both knowledge and attitudes about depression and dementia in older people as well as the quality of support provided to an older person with poor mental health.

**Applications of the study findings**

- The course has the potential to be an effective public health intervention that teaches members of the public how to assist an older person experiencing mental health problems.
- The Older Person MHFA course may fill a gap in evidence-based interventions focusing on older people’s mental health and be particularly beneficial to those without mental health training who are working or volunteering in the aged care industry.

## Introduction

The mental health needs of older people can be complex. Still, they should be addressed as nearly one in ten people over 65 years have an anxiety, affective or substance use disorder in any one year (Australian Bureau of Statistics (ABS), 2023). Additionally, dementia is a neurocognitive disorder with a higher prevalence in this age group (Australian Institute of Health and Welfare, 2023), where early signs can overlap, and be concurrent, with common mental health problems such as depression (Leyhe et al., 2017). Early intervention through increasing mental health literacy in the community, reducing stigma, and facilitating appropriate health care may lead to better outcomes for people with these conditions (Rasmussen & Langerman, 2019; Rewerska-Juśko & Rejdak, 2020). Mental Health First Aid® (MHFA®) is an early intervention program for the public that has been shown to increase knowledge and skills and reduce stigma related to mental health problems (Morgan et al., 2018).

MHFA courses teach participants how to help someone who is experiencing poor or worsening mental health, or a mental health crisis. First developed in 2000 in Australia, MHFA courses have been delivered to over 7 million people worldwide in 29 countries (MHFA, 2024). The Standard MHFA course teaches participants about a range of mental health problems including depression, anxiety, psychosis, and substance use problems, as well as mental health crises such as suicidality and panic attacks. Participants are taught the MHFA Action plan which has six components: 1) Approach the person, 2) Assess and assist with any crisis, 3) Listen and communicate non-judgementally, 4) Give support and information, 5) Encourage appropriate professional help, and 6) Encourage other supports (Kitchener & Jorm, 2002). Since then, the suite of MHFA courses has diversified to include short courses on specific mental health conditions, for example, problem gambling, and courses about specific population groups, for example, Youth MHFA. Previous evaluations of MHFA courses have consistently shown improved knowledge about mental health problems, decreased personal stigma and social distance, increased quality of helping behaviors, and confidence in helping a person (Morgan et al., 2018). The Standard MHFA course, however, does not cover topics relating specifically to older people.

In 2017, Mental Health First Aid Australia launched the Older Person MHFA course, in which the curriculum covered the topics in the Standard MHFA course but the information was tailored to be relevant to the mental health of older people (over 65 years) and also included the topics of confusion, dementia, and delirium (Kitchener et al., 2017). Additional guidelines were also created to inform the course. These drew on the expertise of 65 professionals and carer advocates, who endorsed 389 items on the knowledge, skills, and actions needed to assist someone who is developing cognitive impairment or has dementia or delirium (Bond et al., 2016). This study aimed to evaluate the effectiveness of the Older Person MHFA course. We hypothesized that the course would improve participants’ knowledge, confidence, willingness to help, stigmatizing attitudes, quality of intended helping behaviors, and quality of actual helping behaviors.

## Methods

### Intervention

The Older Person MHFA course is a 12-h educational program that teaches about mental health problems (depression, anxiety, dementia, psychosis, and substance use problems) and mental health crises (suicidal thoughts and behaviors, panic attacks, delirium, and unsafe and challenging behaviors due to confusion) among people over the age of 65. The course is taught by a licensed instructor trained by Mental Health First Aid Australia. The course materials include a PowerPoint slide presentation, handouts, the Older Person MHFA manual, videos, and interactive activities. The course’s learning objectives are to (1) gain an understanding of the prevalence, risk factors and impact of common mental health problems in older Australians; (2) gain an understanding of interventions to support recovery from mental health problems; (3) learn about MHFA and the MHFA Action Plan (ALGEE); (4) learn possible signs, symptoms, and interventions for depression and anxiety problems, suicidal ideation and behavior, panic attacks, confusion and dementia, delirium and unsafe behaviors, psychosis and acute psychotic episodes, and substance use problems; and (5) learn to apply the MHFA Action Plan to the above mental health problems.

### Participants

Participants were eligible if they were attending an Older Person MHFA course, were at least 18 years of age, and consented to participate. Based on effect sizes of mental health first aid intentions in previous uncontrolled trials of MHFA courses (e.g., Bond et al., 2015), a power analysis indicated that 64 participants would give 80% power to detect a medium effect size (d = 0.5) from pre- to post-test, with alpha = 0.05, and conservatively assuming no correlation between pre- and post-training scores. The required sample size of n = 64 was increased to n = 100 to allow for attrition between baseline and follow-up.

### Procedures

Study participants were recruited from Older Person MHFA courses that were conducted in Australia between 2017 and 2022. This lengthy recruiting period was partly due to the impact of the COVID-19 pandemic and related lockdown. A researcher from Mental Health First Aid Australia contacted MHFA Instructors for permission to collect evaluation data from courses that were being conducted at locations that were accessible to the research team. At the beginning of each course, the researcher invited course attendees to participate in the study. Those who were interested were given a Plain Language Statement, consent form, and a copy of the pre-course survey. Participants were also invited to take part in the study via an email distributed by their course instructor inviting them to complete the survey online via the survey Web site SurveyMonkey. Participants were approached again by the researcher immediately after the course to complete the post-course survey and were contacted via email and telephone to complete the six-month follow-up survey, see Supplemental File 1. This project was approved by the Human Research Ethics Committee of the University of Melbourne.

## Measures

### Demographics

Participants were asked their age, sex, Aboriginal and/or Torres Strait Islander status, language spoken at home, highest level of education, and the reason why they were doing the course, that is, for their friends/family, work, for myself, just interested, or other.

### Vignettes

In each survey, participants were given two vignettes describing a mental health problem consistent with DSM-IV diagnostic criteria and asked a series of questions about each one. The depression vignette, describing “John,” has been used extensively in previous research (Kitchener & Jorm, 2002). In this study, John’s age was adjusted to 70 years old. The dementia vignette was taken from Chong et al. (2016) and adapted to describe a 75-year-old named “Paula” (see Supplemental File 1).

### Recognition of Depression or Dementia

Participants were asked an open-ended question about what they thought was wrong with John and Paula. Responses were coded by researchers blinded to time point and participant identification number (Gwet’s agreement coefficient depression = 0.92, dementia = 0.97, N = 35). Responses for the depression vignette were considered correct if the participant identified that John was experiencing depression. For the dementia vignette, responses were coded as correct if they identified that Paula was experiencing dementia or prominent symptoms of dementia such as cognitive changes, memory loss, or confusion.

### Willingness to Help Someone Experiencing Depression or Dementia

Participants were asked to rate their willingness to help John and Paula by responding to the statement “If John/Paula was someone I knew and cared about I would help them” on a 5-point scale ranging from 1 (“strongly disagree”) to 5 (“strongly agree”).

### Quality of Intended Helping Behaviors

Participants were asked what they would do to help someone like John or Paula. Responses were recorded in an open textbox and coded according to a scoring framework based on the MHFA Action Plan used in previous research (Reavley et al., 2018). The scoring criteria are based on the six aforementioned components which can be scored as 0 points (absent), 1 point for a superficial or partial answer (e.g., “Talk to the person”) or 2 points for a detailed, specific answer (e.g., “Listen empathetically”). Total scores representing the quality of the actions were used as the unit of analysis and ranged from 0 to 12. In this study, the MHFA Action Plan framework was adapted to someone experiencing dementia. Examples of these adaptations included delirium as a crisis scenario, suggesting the person see a geriatrician or other older person-specific medical professional, and adopting appropriate listening behaviors for people experiencing confusion (see Supplemental File 2). All answers were scored by two researchers (who are authors of the course), blinded to timepoint and participant ID, and agreement was met for each score.

### Confidence in Intended Helping Behaviors

Participants were asked to rate how confident they would be to help John or Paula on a Likert scale ranging from 1 (“not at all confident”) to 5 (“extremely confident”). This item has shown sensitivity to MHFA training effects in previous evaluations and test-retest reliability over a 2-month period of r = 0.64 (Reavley et al., 2018).

### Stigmatizing Attitudes

The Personal Stigma Scale was adapted from Yap et al. (2014) and used to measure stigmatizing attitudes about depression at each time point. Participants were asked to rate how strongly they agreed or disagreed with seven statements about John on a Likert scale ranging from 1 (“strongly disagree”) to 5 (“strongly agree”). The Personal Stigma Scale loads onto two factors each with four items: dangerous/unpredictable and weak-not-sick (omega = 0.90 and 0.67, respectively). Mean scores were generated for each factor if at least 80% of all statements were rated. Possible scores for each factor ranged from 1 (low stigma) to 5 (high stigma).

This scale was also used for the Paula vignette. As this scale has not previously been used to measure personal stigma toward dementia, a confirmatory factor analysis was conducted to identify its factor structure for dementia stigma. Contrary to Yap et al. (2014), item 5 (“It is best to avoid Paula so that you don’t develop this problem yourself”) loaded onto only the weak-not-sick factor, and not both the weak-not sick and dangerous/unpredictable factor. Satisfactory model fit was achieved (χ2(12) = 16.07, *p* = .188, RMSEA = 0.048, CFI = 0.989, TLI = 0.981, GFI = 0.960, AGFI = 0.929) when allowing for correlated error terms for items 1 (“Paula could snap out of it if she wanted”) and 3 (“Paula’s problem is not a real medical illness”). The standardized loadings for the weak-not-sick factor ranged from 0.65 to 0.86, omega = 0.95 and for the dangerous/unpredictable factor 0.40 to 0.74, omega = 0.71. As above, mean scores were generated for each factor and ranged from 1 (low stigma) to 5 (high stigma).

### Social Distance Scale

We used an adapted version of the Social Distance Scale as a measure of stigma (Link et al., 1999). The Social Distance Scale is a widely used measure and has shown validity in measuring stigma toward people with dementia (Subramaniam et al., 2017). Participants responded to eight questions relating to social contact with a person like John or Paula, such as whether the participant would “Move next door to John” and “Have John as a father-in-law” on a 5-point Likert scale from 1 (“definitely not”) to 5 (“yes, definitely”). Mean scores were generated on reversed scores, such that low values indicate low social distance and high values indicate high social distance (depression omega = 0.93; dementia omega = 0.95).

### Knowledge About Mental Health Problems

At each time point, participants were also given a series of 14 statements about mental health problems based on the Older Person MHFA course content and asked to respond with “agree,” “disagree,” or “don’t know” to determine their level of knowledge about mental health problems in older adults. These were similar to knowledge questions that have been used in previous evaluations of MHFA training (Morgan et al., 2018), but included four questions specifically on confusion or delirium and two questions modified to refer to older adults. Seven statements were considered false (e.g., If an older person who is confused repeats questions or statements over and over, it is best to ignore them) and seven were considered true (e.g., If an older person is experiencing delirium, it is helpful to reduce distracting noises, such as radio and television). Responses of “don’t know” were coded as incorrect. Using exploratory factor analysis, a one-factor model was deemed appropriate based on the scree plot. Two statements were removed from the scale due to collinearity (“Anxiety disorders and depression are less common in older people than young adults”) or inadequate loading (<0.09) (“Most older people with a common mental illness do not get professional help”). The remaining items formed a unidimensional factor with an omega value of 0.75. The knowledge scale was subsequently analyzed as the percentage of correct responses (range 0–100%) if at least 80% of questions were answered. Higher scores indicated greater knowledge about mental health problems.

### Quality of Actual Helping Behaviors

At the end of the pre-course and follow-up survey, participants were asked to recall a time when they knew an older person (aged 65+) over the last six months who had any sort of mental health problem, including dementia. They were then asked about the age, gender, and relationship to the person and what they thought their mental health problem was. Participants were asked whether they had helped the person. If they responded “no,” they were asked to give the reasons why they did not help in a free-text box. If they responded “yes” they were asked to describe all the things they did to help in a free-text box and were coded using a combination of the dementia and depression MHFA Action plan scoring framework of the intended helping behaviors responses. All answers were scored by two researchers (who are authors of the course), blinded to timepoint and participant ID, and agreement was met for each score.

### Course Satisfaction

At the end of the post-course survey, participants were asked to give feedback on the course. They rated the novelty of the content, their understanding of the content, presentation, relevance, and materials of the program on a 5-point Likert scale. They were also asked open-ended questions on what they found most helpful and what could be improved.

### Manual Usage After the Course

In the six-month follow-up survey, participants were asked about the Older Person MHFA manual. They were asked to rate on a 4-point Likert scale how much of the manual they had read, how much they learned, the usefulness of the manual, and whether they would recommend the manual to others. On a 5-point Likert scale, they were also asked how easy the manual was to read. Further, they were asked what they did with the manual and whether they would use it in the future in a free-text box.

### Statistical Analysis

Data were analyzed using linear and logistic mixed models. Models included a fixed effect of time and a random effect of participants and course to adjust for the correlation of responses within participants and within courses over time. Models retained all available data and provide an intention-to-treat estimate of change over time under the missing-at-random assumption. Using logistic regression models, missingness was investigated to determine whether any demographic variables (age, gender, language spoken at home, Aboriginal or Torres Strait Islander status, education, and reason for doing the course) or pre-course outcomes were associated with data missing at six-month follow-up. Age and speaking a language other than English at home were associated with missingness at follow-up and were included as fixed effects to help meet the missing-at-random assumption. Planned comparisons investigated the change over time from pre-course to post-course and pre-course to six-month follow-up.

Two variables had highly skewed distributions and were therefore dichotomized and analyzed with a mixed logistic regression model as a linear model was inappropriate. The weak-not-sick factor of the stigma scale was dichotomized based on scoring 1 (no stigma) or greater than 1 (indicating some stigma) and a logistic regression model was used to determine the odds of scoring 1 (low stigma) after the course. The willingness to help variable was also dichotomized to a score of 5 (highest score) or less than 5 (some unwillingness to help) and analyzed using a mixed logistic regression model.

Effect sizes were calculated and interpreted using Cohen’s d and Cohen’s criteria for small, medium, and large effect sizes, in which the difference between the means was divided by their pooled standard deviation. Analyses were performed using Stata 17 and the significance level was set at *p* < .05.

Open-ended questions were analyzed using content analysis to identify prominent ideas in the data, supplemented with the frequency of each category as a percentage of the total responses.

## Results

### Participant Characteristics

**Figure 1.**
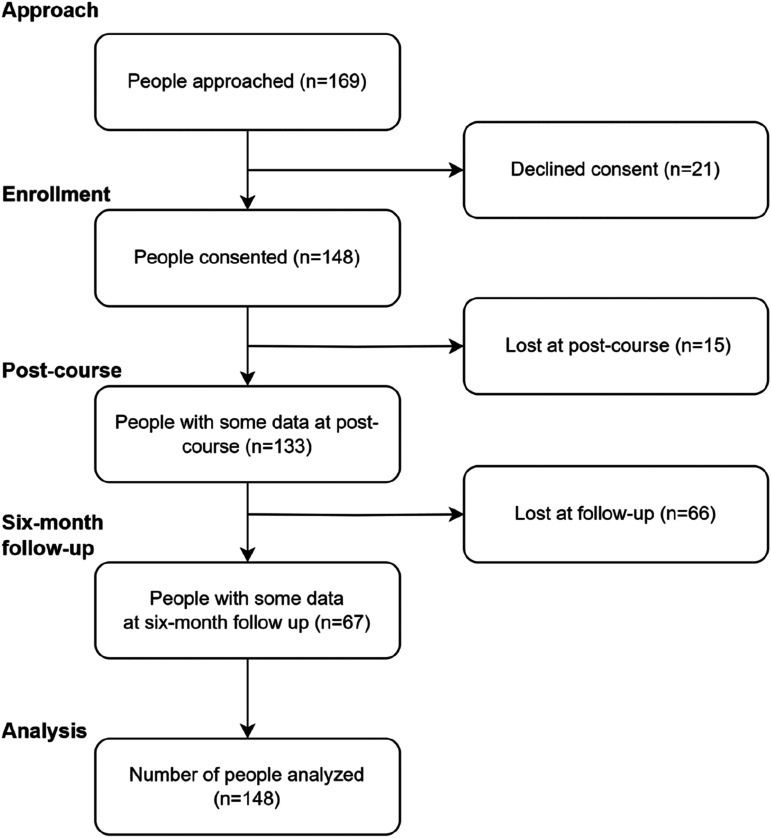
CONSORT flow diagram.

Between 2017 and 2022, 169 participants from 13 courses were approached to participate in the Older Person MHFA course evaluation. Of these, 148 (87.5%) consented to participate and completed the baseline questionnaire, of which, 133 (89.9%) provided some data at the post-course survey, and 67 (45.3%) provided some data at six-month follow-up. Subsequently, data from a total of 148 participants were analyzed, see Figure 1. Predictors of missing data at follow-up were explored, with age and language spoken at home as the only significant predictors associated with missingness. For each additional year of age, the odds of completing the survey increased by 3% (OR 1.03, *p* = .030). Participants who spoke a language other than English at home were significantly less likely to complete the follow-up survey (OR 0.17, *p* = .023).

### Baseline Demographics

Baseline demographics are reported in Table 1. Participants had a mean age of 53 years, the majority were female, and the highest level of education was most commonly a certificate or apprenticeship. The most common reason for participating in the course was for work, followed by for friends or family. One participant (0.68%) did not report any demographic data.

**Table 1.** Participant Demographics (N = 148).

Demographic		N (%)
Age—M(SD)		53 (12.27)
Gender	Male	15 (10.14)
	Female	132 (89.19)
	Missing	1 (0.68)
Education	Some high school	3 (2.03)
	Year 10, 11, or 12	20 (13.51)
	Certificate or apprenticeship	64 (43.24)
	University	60 (40.54)
	Missing	1 (0.68)
Aboriginal and/or Torres Strait Islander status	Aboriginal	2 (1.36)
Language other than English		14 (9.46)
Reasons for doing the course^a^		
For work		117 (79.05)
For family or friends		99 (67.89)
For myself		81 (54.73)
For other people		79 (53.38)
Just interested		15 (10.14)

N, number; M, mean; SD, standard deviation.

^a^Multiple reasons could be selected.

### Disorder Recognition

#### Depression Vignette

A high percentage of participants correctly recognized that John is experiencing symptoms of depression (n = 133, 93.66% at pre-course). There was no significant change in recognition at post-course or six-month follow-up, see Table 2.

**Table 2.** Observed Proportions and Change in Odds Over Time for Binary Outcome Measures.

	Observed Proportions—N (%)			Pre-Course to Post-Course			Pre-Course to Six-Month Follow-Up		
	Pre-Course	Post-Course	Six-Month Follow-Up	Odds Ratio	95% CI	*p*-Value	Odds Ratio	95% CI	*p*-Value
Recognition of depression	133/142 (93.66)	114/123 (92.68)	64/67 (95.52)	0.88	0.28, 2.73	0.822	1.74	0.34, 8.89	0.505
Recognition of dementia	137/146 (93.84)	121/124 (97.58)	63/65 (96.92)	4.86	0.69, 34.20	0.112	1.25	0.16, 9.85	0.832
Willingness to help—depression^a^	111/146 (76.03)	116/132 (87.88)	50/67 (74.63)	3.21	1.43, 7.23	**0.005**	0.85	0.36, 1.96	0.695
Willingness to help—dementia^a^	98/146 (67.12)	103/132 (78.03)	42/65 (64.62)	2.43	1.22, 4.85	**0.011**	0.98	0.44, 2.17	0.961
Stigma weak/not sick factor—depression^b^	73/144 (50.69)	94/132 (71.21)	44/67 (65.67)	4.28	2.11, 8.67	**<0.001**	2.70	1.19, 8.67	**0.018**
Stigma weak/not sick factor—dementia^b^	100/145 (68.97)	103/131 (78.63)	52/65 (80.00)	2.85	1.32, 6.05	**0.007**	3.13	1.16, 8.42	**0.024**

Bold values indicate statistically significant results.

^a^Scores dichotomized to 5 (strongly agree) versus <5.

^b^Scores dichotomized to 1 (no stigma) versus >1 (some stigma).

#### Dementia Vignette

Dementia was highly recognized by participants at pre-course (n = 137, 93.84%), post-course (n = 121, 97.58%), and at six-month follow-up (n = 63, 96.92%). These changes were not significant, see Table 2.

### Willingness to Help

#### Depression Vignette

At pre-course 76% (n = 111) of participants responded with “strongly agree” with the statement that they would help someone like John, which increased significantly at post-course course (OR = 3.21, *p* = .005) but not at six-month follow-up (OR = 0.85, *p* = .695; see Table 2).

#### Dementia Vignette

Ninety-eight participants (67.1%) reported that they “strongly agree,” which increased significantly at post-course (OR = 2.43, *p* = .011) but these results were not significant at six-month follow-up (OR = 0.98, *p* = .961), see Table 2.

### Quality of Intended Helping Behaviors

#### Depression Vignette

Participants’ mean score for quality of intended helping behaviors was 4.01 (SD = 1.48) at pre-course (see Table 3). Quality of intended behaviors increased significantly at post-course (M = 1.66, *p* < .001), which was a large effect (d = 0.93) but was not significant at six-month follow-up, see Table 4.

**Table 3.** Observed Mean and Standard Deviations of Linear Outcome Measures at Each Time Point.

	Pre-Course	Post-Course	Six-Month Follow-Up
Depression vignette—N, M (SD)			
Confidence in helping^a^	146, 3.25 (1.00)	129, 4.20 (0.68)	67, 3.93 (0.74)
Stigma (dangerous/unpredictable)^a^	145, 1.91 (0.62)	131, 1.75 (0.83)	67, 1.77 (0.58)
Social distance^a^	127, 2.03 (0.76)	131, 1.88 (0.76)	58, 1.81 (0.67)
Quality of intended helping behaviors (Action Plan score)^b^	140, 4.01 (1.48)	123, 5.68 (2.12)	67, 4.23 (1.96)
Dementia vignette—N, M (SD)			
Confidence in helping^a^	145, 3.43 (1.04)	132, 4.29 (0.63)	65, 3.94 (0.85)
Stigma (dangerous/unpredictable)^a^	143, 2.28 (0.84)	129, 1.95 (0.96)	65, 2.18 (0.78)
Social distance^a^	121, 1.97 (0.77)	130, 1.82 (0.75)	56, 1.74 (0.63)
Quality of intended helping behaviors (Action Plan score)^b^	140, 3.41 (1.28)	121, 5.00 (1.81)	65, 4.09 (2.17)
Knowledge about Mental Health Problems, per cent correct^c^ N, M (SD)	145, 60.02 (18.85)	130, 83.49 (11.69)	65, 78.97 (12.25)
Quality of actual helping behaviors (Action Plan score)^b^ N, M (SD)	79, 2.77 (1.66)	N/A	39, 3.64 (1.83)

^a^Range 1–5.

^b^Range 0–12.

c Range 0–100.

**Table 4.** Mean Change Over Time of Linear Outcome Measures.

Outcome	Pre-Course to Post-Course					Pre-Course to Six-Month Follow-Up				
	Mean Change^a^	95% CI	*p*-Value	Effect Size^a^	95% CI	Mean Change^a^	95% CI	*p*-Value	Effect Size^a^	95% CI
Quality of intended helping behaviors (Action plan score)—depression vignette^a,b^	1.66	1.25. 2.07	**<0.001**	0.93	0.67, 1.18	0.24	−0.27, 0.74	0.449	0.14	−0.15, 0.43
Quality of intended helping behaviors (Action plan score)—dementia vignette	1.61	1.23, 1.99	**<0.001**	1.03	0.77, 1.29	0.70	0.23, 1.17	**0.004**	0.42	0.13, 0.72
Confidence in helping—depression vignette	0.98	0.82, 1.14	**<0.001**	1.10	0.84, 1.35	0.69	0.49, 0.89	**<0.001**	0.72	0.75, 1.25
Confidence in helping—dementia vignette	0.89	0.72, 1.05	**<0.001**	1.00	0.75, 1.25	0.54	0.33, 0.75	**<0.001**	0.52	0.22, 0.82
Stigma (dangerous/unpredictable)—depression vignette	−0.18	−0.32, −0.03	**<0.001**	−0.22	−0.46, 0.02	−0.17	−0.36, 0.01	0.060	−0.23	−0.52, 0.06
Stigma (dangerous/unpredictable)—dementia vignette	−0.36	−0.54, −0.19	**<0.001**	−0.35	−0.59, −0.11	−0.17	−0.39, 0.05	0.136	−0.12	−0.41, 0.18
Social distance—depression vignette	−0.18	−0.31, −0.05	**0.005**	−0.19	−0.44, 0.05	−0.22	−0.39, −0.05	**0.013**	−0.30	−0.62, 0.01
Social distance—dementia vignette	−0.20	−0.31, −0.09	**<0.001**	−0.20	−0.44, 0.05	−0.23	−0.37, −0.08	**0.002**	−0.32	−0.64, 0.002
Knowledge about mental health problems	23.67	20.51, 26.83	**<0.001**	1.48	1.21, 1.75	19.36	15.35, 23.38	**<0.001**	1.11	0.80, 1.42
Quality of actual helping behaviors (Action plan score)^b,c^	N/A	N/A	N/A	N/A	N/A	0.97	0.32, 1.61	**0.005**	0.51	0.16, 0.89

95% CI, 95% confidence interval; N/A, not applicable.

^a^Negative values indicate a decrease in the outcome measure, positive values indicate an increase in the outcome measure.

^b^This variable was transformed due to the non-normal distribution of model residuals.

^c^Based on 118 responses.

#### Dementia Vignette

The mean score for quality of intended behavior at pre-course was 3.41 (SD = 1.28; see Table 3). Contrary to the depression vignette, mean change over time was significant at post-course (M = 1.72, *p* < .001), and six-month follow-up (M = 0.70, *p* = .004) with a large effect size (see Table 4).

### Confidence

#### Depression Vignette

At baseline, participants were moderately confident in helping someone with a problem like John’s (M = 3.25, SD = 1.0), see Table 3, and this increased significantly over time at both post-course (M = 0.98, *p* < .001) and six-month follow-up (M = 0.69, *p* < .001), see Table 4.

#### Dementia Vignette

Similar findings were observed about helping a person with a problem like Paula’s. Confidence increased significantly at post-course (M = 0.89, *p* < .001) and six-month follow-up (M = 0.54, *p* < .001), see Table 3. Effect sizes were large at post-course but moderate-to-large at follow-up.

### Personal Stigma

#### Depression Vignette

Belief in dangerousness/unpredictability was low at pre-course (M = 1.91, SD = 0.56), see Table 3. There was a significant change at post-course (M = −0.18, *p* < .001) but not at six-month follow-up, see Table 4. Conversely, weak-not-sick beliefs showed a significant decrease over time at post-course (OR = 4.28, *p* < .001) and six-month follow-up (OR = 2.70, *p* = .018), see Table 2.

#### Dementia Vignette

Similar to the depression vignette, weak-not-sick beliefs were lower (M = 1.29, SD = 0.59) than dangerous/unpredictability beliefs at pre-course (M = 2.28, SD = 0.84), see Table 3. Weak-not-sick stigma decreased significantly at both post-course (OR = 2.85, *p* = .007) and six-month follow-up (OR = 3.13, *p* = .024), see Table 2. Mean change in beliefs about dangerousness/unpredictability decreased significantly at post-course (M = −0.36, *p* < .001), but not at six-month follow-up, and a moderate effect size was observed at post-course (d = −0.35), see Table 4.

### Social Distance

#### Depression Vignette

Desire for social distance from a person with depression was low before the course (M = 2.03 SD = 0.76, see Table 3), yet there was still a significant decrease at both post-course (M = −0.18, *p* = .005) and six-month follow-up (M = −0.22, *p* = .013) when compared to pre-course, see Table 4. A small effect size was observed at both time points.

#### Dementia Vignette

Desire for social distance from a person with dementia was also low at pre-course (M = 1.97 SD = 0.77), see Table 3. There were small but significant decreases in social distance over time at post-course (M = −0.20, *p* < .001) and six-month follow-up (M = −0.23, *p* = .002).

### Knowledge About Mental Health problems

The mean knowledge score at pre-course was 60.02% (SD = 18.85; see Table 3), which increased significantly over time at both post-course (M = 23.67, *p* < .001) and six-month follow-up (M = 19.36, *p* < .001). Large effect sizes were observed when comparing pre-course to post-course estimates (d = 1.49), and pre-course to six-month follow-up estimates (d = 1.13), see Table 4.

### Quality of Actual Helping Behaviors

At pre-course, 108 people (70.27%) responded that they knew someone over the age of 65 who had a mental health problem, including dementia, and most of these reported providing help (see Table 5). Results were similar at follow-up. Thirty-three participants reported helping someone at both time points.

**Table 5.** Characteristics of the People Helped by Participants in the Course.

	Pre-Course—N (%)	Six-Month Follow-Up—N (%)
Participants who knew anyone (over 65) who had a mental health problem	108 (70.27)^a^	42 (62.7)^b^
How many people^c^		
Know more than one person^c^	80 (74.1)	28 (66.7)
Just one^c^	23 (21.3)	10 (23.8)
I’d rather not say	3 (2.8)	4 (9.5)
Missing	2 (1.9)	0
Age of the person^c^		
65–69	29 (26.9)	5 (11.9)
70–79	33 (30.5)	15 (35.7)
80+	40 (37)	18 (42.9)
Missing	6 (5.6)	4 (9.5)
Gender of the person^c^		
Male	44 (40.7)	15 (35.7)
Female	57 (52.8)	23 (54.8)
Don’t know	1 (0.9)	0
Missing	6 (5.6)	4 (9.5)
Relationships^c^		
Family	40 (38.8)	10 (23.8)
Friend	18 (17.4)	6 (14.3)
Client	37 (33.9)	22 (52.4)
Colleague	3 (2.9)	0
Other	4 (5.8)	0
Missing	6 (5.6)	4 (9.5)
Did they help the person^c^		
Yes	87 (80.6)	37 (88.1)
No	16 (14.8)	1 (2.4)
Missing	5 (4.6)	4 (9.5)

^a^Percentage of people who had data at pre-course.

^b^Percentage of people who had data at six-month follow-up.

^c^Percentage of people who knew someone with a mental health problem.

On average, the quality of helping behavior at baseline was M = 2.77 (SD = 1.66), see Table 3. At six-month follow-up, there was a significant increase in participants’ scores (M = 0.97, *p* = .005), see Table 4. This effect size was moderate-to-large (d = 0.66).

At baseline and follow-up, only 13 participants gave reasons for not providing help. As such, these responses were not analyzed.

### Course Satisfaction and Manual Use

Overall, participants perceived the course to be acceptable. Participants gave high ratings to the manual (M = 4.9, SD = 0.33), PowerPoint (M = 4.73, SD = 0.55), films (M = 4.88, SD = 0.38), and activities (M = 4.67, SD = 0.65) on a 1 (“not very much”) to 5 (“very much”) Likert scale. At six-month follow-up, the manual was also highly rated by participants, with 80% reporting that they would definitely recommend the manual to others and 87.3% reporting that they would use the manual in the future. Further course satisfaction and manual usage data are presented in Supplemental File 3.

## Discussion

This evaluation of the Older Person MHFA course showed improvements in knowledge about mental health problems, reductions in both desire for social distance and personal stigma toward people with depression or dementia, and increased confidence and willingness to help someone experiencing depression or dementia, with many effects sustained six months after training. Most importantly, course participants demonstrated a significant improvement in the quality of their intended helping behaviors toward someone experiencing depression or dementia and an increase in the quality of help provided to people they knew. The Older Person MHFA course was also highly acceptable to participants.

Although the quality of intended help toward someone experiencing depression increased significantly post-course, this was not maintained at six-month follow-up. However, there was sustained improvement in the quality of intended help toward a person experiencing dementia at six months. While the latter result aligned with other MHFA course evaluations in which increases in the quality of helping intentions were sustained at six months (Morgan et al., 2018), it doesn’t align with the former result regarding depression. It is possible that participants may not have provided exhaustive lists of everything they would do or had done for the person as these were coded free-text responses. Future evaluations of the Older Person MHFA course should consider adopting the Mental Health Support Scale, a newly validated and reliable measure of MHFA behaviors that does not use free-text response options (Morgan et al., 2023). Contrary to other MHFA evaluations which have shown a lack of statistical power for detecting effects on helping behavior (Forthal et al., 2022), this study found that a majority of participants had known someone with a mental health problem in the past six months and that there was a significant increase in the quality of helping actions to someone experiencing a mental illness outside of the course.

Uniquely, the Older Person MHFA course includes learning the possible signs, symptoms, and interventions for dementia. Participants showed high levels of recognition of dementia, which contrasts with a representative survey of the Australian public that found knowledge about dementia is poor (Kim et al., 2023). The majority of the study sample reported that they were doing this course for work and may therefore already have prior knowledge of dementia. Perhaps these participants were seeking more information on how to help someone they know rather than how to recognize when a person may have dementia. Despite high levels of recognition, participants showed improved confidence in helping and lower stigmatizing attitudes toward a person with dementia after the course. These positive effects are important because high confidence to help and low stigma have been identified as important predictors of providing high-quality support to people with mental health problems (Usmani et al., 2023). Stigmatizing attitudes toward people with mental health problems have also been identified as a barrier to professional help-seeking in older people (Elshaikh et al., 2023) particularly for dementia (Rewerska-Juśko & Rejdak, 2020; Zhang et al., 2021).

Finally, comparisons can be made between this course and a Swedish adaptation of the Standard MHFA course to help the elderly, which also observed an increase in knowledge and quality of helping behaviors, as well as improvements in stigmatizing attitudes with a small to moderate effect size (Svensson & Hansson, 2014). However, comparisons are limited due to methodological differences in the scales used between these studies. It is difficult to compare the Older Person MHFA course to other early intervention mental health programs because of its distinct approach to addressing older people’s mental health needs. Other mental health programs often target nurses working in aged care to improve the mental health of older people, such as nurse-led programs to reduce depression (Almeida et al., 2022; Markle-Reid et al., 2014). Mental health programs have also frequently focused on reducing social isolation as a mechanism to improve psychological well-being and reduce depression and anxiety (Lee et al., 2022). The Older Person MHFA program differs as the broad target audience includes, but is not limited to, professionals, and it focuses on mental health literacy. These results suggest that the Older Person MHFA course may be well placed to fill a gap when addressing the mental health needs of older people.

### Strengths and Limitations

This is the first time that an MHFA course has introduced and assessed the topic of dementia, filling a gap in the MHFA suite of courses. A further strength of the Older Person MHFA course is the design and delivery. A recent study surveyed the preferred format of mental health information among older people and found that written information, for example, a pamphlet, was preferred over the Internet (Reynolds et al., 2023). This preference is noteworthy because the mean age in this sample is higher than that of other MHFA course evaluations, suggesting that young/middle-aged people are attending to learn how to help older people, and that older people are attending to learn how to help their peers. The study also had a very high participation rate, which suggests minimal selection bias, and excellent retention at post-course. This study also included a contemporary investigation of what participants do with their MHFA manual, which indicated that it is a useful resource for participants after the course.

There are also limitations in this study. The absence of a control group makes it challenging to discern the true effectiveness of the Older Person MHFA course. While the retention rate was high post-course, the number of participants retained at six-month follow-up dropped to less than half, possibly leading to a lack of power to detect small effects. Further, the sample was mostly female and well-educated (as is typical in evaluations of MHFA courses (Morgan et al., 2018)) and findings may not be generalized to other population groups, such as less-educated males.

### Future Directions

Future evaluations of the Older Person MHFA course should consider using a randomized controlled trial design to provide a robust assessment of the effectiveness of the course. Additionally, establishing how long benefits are sustained, by including long-term (e.g., up to 2 years) follow-up would provide useful insights into long-term effectiveness. Researchers should also consider implementing the program within specific population groups that may be at higher risk of developing dementia, such as aged care home facilities and retirement villages, and within community programs that interact with older people, such as “Meals on Wheels.”

## Conclusion

This study found that the Older Person MHFA course may be an effective early intervention at reducing the desire for social distance and personal stigma toward people with depression and dementia and increasing knowledge about common mental health problems in older people, confidence, and willingness to help and the quality of both intended and actual helping behaviors. It may be of particular benefit to those without mental health training who are working or volunteering in the aged care industry. Further research is needed to determine the effectiveness compared to a control intervention, how long-lasting the effects are, and whether the course provides benefits to the recipients of MHFA support (Richardson et al., 2023).

## Supplemental Material

Supplemental Material - Evaluation of the Older Person Mental Health First Aid Course: Effects on Knowledge, Stigmatizing Attitudes, and Helping Behaviors Supplemental Material for Evaluation of the Older Person Mental Health First Aid Course: Effects on Knowledge, Stigmatizing Attitudes, and Helping Behaviors by Julia Lyons, Kathy Bond, Betty Kitchener, Fairlie Cottrill, Anthony Jorm, Alyssia Rossetto, Claire Kelly, Nicola Reavley, and Amy Morgan in Journal of Applied Gerontology.

Supplemental Material - Evaluation of the Older Person Mental Health First Aid Course: Effects on Knowledge, Stigmatizing Attitudes, and Helping Behaviors Supplemental Material for Evaluation of the Older Person Mental Health First Aid Course: Effects on Knowledge, Stigmatizing Attitudes, and Helping Behaviors by Julia Lyons, Kathy Bond, Betty Kitchener, Fairlie Cottrill, Anthony Jorm, Alyssia Rossetto, Claire Kelly, Nicola Reavley, and Amy Morgan in Journal of Applied Gerontology.

Supplemental Material - Evaluation of the Older Person Mental Health First Aid Course: Effects on Knowledge, Stigmatizing Attitudes, and Helping Behaviors Supplemental Material for Evaluation of the Older Person Mental Health First Aid Course: Effects on Knowledge, Stigmatizing Attitudes, and Helping Behaviors by Julia Lyons, Kathy Bond, Betty Kitchener, Fairlie Cottrill, Anthony Jorm, Alyssia Rossetto, Claire Kelly, Nicola Reavley, and Amy Morgan in Journal of Applied Gerontology.

## Data Availability

The datasets used and/or analyzed during the current study are available from the corresponding author upon reasonable request.*
