# Supplementary material for: Evaluation of the Older Person Mental Health First Aid Course: Effects on Knowledge, Stigmatizing Attitudes, and Helping Behaviors
Source: J Appl Gerontol. 2025 Jul 8;45(5):856–67. doi: 10.1177/07334648251352309 (PMC13076979; doi:10.1177/07334648251352309)
Supplement: Supplemental Material - Evaluation of the Older Person Mental Health First Aid Course: Effects on Knowledge, Stigmatizing Attitudes, and Helping Behaviors [file sj-pdf-1-jag-10.1177_07334648251352309.pdf]

Evaluating the Mental Health First Aid course for the Older Person

(Survey T1)

1. **Please provide us with your name and email address.** (We collect this data in order to match your surveys. Once your surveys are matched, your name is deleted from the information in the survey. Only the research officers have access to identifying information, we **do not** share any identifying information/data with your employers, colleagues, community members, etc.).

Name

Email

Phone number

2. **What is your gender**?

☐ Male

☐ Female

☐ I identify with another term

3. **How old are you** (in years)?

4. **What is the highest level of education you have completed**?

☐ Primary school

☐ Some high school

☐ Years 10, 11, OR 12

☐ Certificate, Trade OR Apprenticeship

☐ University

5. **Are you:**

☐ Aboriginal

☐ Torres Strait Islander

☐ Both

☐ Neither

6. **Do you speak a language other than English as your first language at home?**

☐ Yes

☐ No

7. **Why are you interested in learning Mental Health First Aid** (tick as many as necessary)?

☐ To assist people in my workplace

☐ To assist family or friends

☐ To look after my own mental health

☐ I want to learn to help other people

☐ Just interested – no particular reason

☐ Other

The following section concerns a hypothetical person called John. The description below outlines how he has been recently.

John is 70 years old. He has been feeling unusually sad and miserable for the last few weeks. Even though he is tired all the time, he has trouble sleeping nearly every night. John doesn't feel like eating and has lost weight. He can't keep his mind on things and puts off making decisions. Even day-to-day tasks seem too much for him. John's wife is concerned about the changes she has seen in him. John feels he will never be happy again and believes his family would be better off without him. John has been so desperate, he has been thinking of ways to end his life.

8. **What, if anything, do you think is wrong with John**?

9. **If John was someone I knew and cared about, I would help him.** (Circle the number that represents your response to this statement.)

1<br>Strongly disagree	2	3	4	5<br>Strongly agree

10. **Describe all the things you would do to help John**.

11. **How confident do you feel in helping someone with a problem like John's?** (Circle the number that represents your answer.)

1<br>Not at all confident	2	3	4	5<br>Extremely confident

12. **The next few questions contain statements about John's problem.** (Please indicate how strongly you agree or disagree with each statement.)

	Strongly disagree	Disagree	Neither agree nor disagree	Agree	Strongly Agree
John could snap out of it if he wanted.					
John's problem is a sign of personal weakness.					
John's problem is not a real medical illness.					
John is dangerous.					
It is best to avoid John so that you don't develop this problem yourself.					
John's problem makes him unpredictable.					
I would not tell anyone if I had a problem like John's.					

13. **The following questions ask how you would feel about spending time with John.**
Would you be happy to...

	Definitely not	Probably not	Not sure	Yes, probably	Yes, definitely
Move next door to John?					
Spend an evening socializing with John?					
Make friends with John?					
Go to John's house?					
Invite John around to your house?					
Work or volunteer with John?					
Have a close relationship with John?					
Have John as a father-in-law?					

The following section concerns a hypothetical person called Paula. The description below outlines how she has been recently.

Paula is 75 years old and retired. Her husband has noticed that she has problems remembering things that happened recently but recalls things from earlier in their marriage quite well. She repeats questions which she has already answered, misplaces her things and occasionally gets confused during their conversations. Sometimes Paula and her husband quarrel as she accuses him of taking her things. She lost her way once or twice whilst driving to their son's home, and has written some cheques for the wrong amount when paying bills. When her husband points out these problems to Paula, she loses her temper. She does not think she has a problem.

14. **What, if anything, do you think is wrong with Paula**?

15. **If Paula was someone I knew and cared about, I would help her.** (Circle the number that represents your response to this statement.)

1<br>Strongly disagree	2	3	4	5<br>Strongly agree

16. **Describe all the things you would do to help Paula**.

17. **How confident do you feel in helping someone with a problem like Paula's?** (Circle the number that represents your answer.)

1<br>Not at all confident	2	3	4	5<br>Extremely confident

18. **The next few questions contain statements about Paula's problem. Please indicate how strongly you agree or disagree with each statement.** (Please indicate how strongly you agree or disagree with each statement.)

	Strongly disagree	Disagree	Neither agree nor disagree	Agree	Strongly Agree
Paula could snap out of it if she wanted.					
Paula's problem is a sign of personal weakness.					
Paula's problem is not a real medical illness.					
Paula is dangerous.					
It is best to avoid Paula so that you don't develop this problem yourself.					
Paula's problem makes her unpredictable.					
You would not tell anyone if you had a problem like Paula's.					

19. **The following questions ask how you would feel about spending time with Paula. Would you be happy to...**

	**Definitely not**	**Probably not**	**Not sure**	**Yes, probably**	**Yes, definitely**
Move next door to Paula?					
Spend an evening socializing with Paula?					
Make friends with Paula?					
Go to Paula's house?					
Invite Paula around to your house?					
Work or volunteer with Paula?					
Have a close relationship with Paula?					
Have Paula as a mother-in-law?					

20. **The next section contains statements about health problems.** Please indicate whether you agree or disagree with each statement, or if you don't know.

Anxiety disorders and depression are less common in older people than young adults.	DISAGREE	AGREE	DON'T KNOW
Most older people with a common mental illness do not get professional help.	DISAGREE	AGREE	DON'T KNOW
If a person who is depressed does not want to seek professional help, it is important to force them to if you can.	DISAGREE	AGREE	DON'T KNOW
Recovery from anxiety disorders requires facing situations which are anxiety provoking.	DISAGREE	AGREE	DON'T KNOW
If an older person who is confused repeats questions or statements over and over, it is best to ignore them.	DISAGREE	AGREE	DON'T KNOW

20. (cont)

When interacting with a person with psychosis, it is best not to offer them choices of how you can help them because it could add to their confusion.	DISAGREE	AGREE	DON'T KNOW
A good way to help a person with a drug or alcohol problem is to let them know that you strongly disapprove of their substance use.	DISAGREE	AGREE	DON'T KNOW
It is not a good idea to ask someone if they are feeling suicidal in case you put the idea in their head.	DISAGREE	AGREE	DON'T KNOW
It is best to get someone having a panic attack to breathe into a paper bag.	DISAGREE	AGREE	DON'T KNOW
If an older person is experiencing delirium, it is helpful to reduce distracting noises, such as radio and television.	DISAGREE	AGREE	DON'T KNOW
If an older person is unsafe driving due to confusion, it is helpful to keep the person driving longer by acting as a 'co-pilot' (e.g. by giving instruction and directions to the person when they drive).	DISAGREE	AGREE	DON'T KNOW
Loud noises, an over-stimulating environment, or physical clutter may cause agitated behaviour in an older person.	DISAGREE	AGREE	DON'T KNOW
It is best not to try to reason with a person having delusions.	DISAGREE	AGREE	DON'T KNOW
If a person is intoxicated with alcohol, it is not possible to make them sober up more quickly by giving them strong coffee, a cold shower or taking them for a walk.	DISAGREE	AGREE	DON'T KNOW

21. **Over the last 12 months, has any older person (aged 65+) you know had any sort of mental health problem?** *(a "mental health problem" is a cluster of symptoms that affects a person's thinking, emotional state and behaviour, and disrupts the person's ability to work or carry out other daily activities and engage in satisfying personal relationships. The problem lasts for a period of weeks or more. This could include problems involving depression, anxiety, dementia, psychosis, alcohol or drug use, or suicide).*

☐ Yes (Go to question 22)

☐ No (Go to the end of the survey)

☐ Don't know (Go to the end of the survey)

☐ I'd rather not say (Go to the end of the survey)

22. **Did you notice a mental health problem in just one older person or have you seen these problems in more than one person?**

☐ More than one, please specify how many _______ (Go to question 23)

☐ Just one (Go to question 24)

☐ I'd rather not say (Go to the end of the survey)

23. **Because you know more than one older person who had a mental health problem, for the next few questions, we'd like you to THINK ABOUT THE PERSON YOU KNOW BEST**.

24. **How old is that person?** (If you don't know exactly, your best guess is fine.)

☐ 65-69 years old

☐ 70-79 years old

☐ 80 years old or over

25. **Are they**:

☐ Male

☐ Female

☐ They identify with another term

☐ Don't know

26. **Would you describe this person as:**

☐ A family member

☐ A friend

☐ A client at your work

☐ A colleague or previous colleague

☐ Other, please specify: ________________

27. **What do you think their mental health problem was?**

28. **Over the last 12 months, did you try to help the person with this problem?**

☐ Yes (Go to question 30)

☐ No (Go to question 29)

29. **If you didn't help the person, are there any particular reasons that you did not try to help the person with this problem? If so, please describe these reasons.** (You do not need to answer question 30.)

30. **If you did help the person, describe all the things you did to help.**

Thank you for completing this survey!

Evaluating the Mental Health First Aid course for the Older Person

(Survey T2)

1. **Please provide us with your name and email address.** (We collect this data in order to match your surveys. Once your surveys are matched, your name is deleted from the information in the survey. Only the research officers have access to identifying information, we **do not** share any identifying information/data with your employers, colleagues, community members, etc.).

Name

Email

The following section concerns a hypothetical person called John. The description below outlines how he has been recently.

John is 70 years old. He has been feeling unusually sad and miserable for the last few weeks. Even though he is tired all the time, he has trouble sleeping nearly every night. John doesn't feel like eating and has lost weight. He can't keep his mind on things and puts off making decisions. Even day-to-day tasks seem too much for him. John's wife is concerned about the changes she has seen in him. John feels he will never be happy again and believes his family would be better off without him. John has been so desperate, he has been thinking of ways to end his life.

2. **What, if anything, do you think is wrong with John**?

3. **If John was someone I knew and cared about, I would help him.** (Circle the number that represents your response to this statement.)

1<br>Strongly disagree	2	3	4	5<br>Strongly agree

4. **Describe all the things you would do to help John**.

5. **How confident do you feel in helping someone with a problem like John's?** (Circle the number that represents your answer.)

1<br>Not at all confident	2	3	4	5<br>Extremely confident

6. **The next few questions contain statements about John's problem.** Please indicate how strongly you agree or disagree with each statement.

	Strongly disagree	Disagree	Neither agree nor disagree	Agree	Strongly Agree
John could snap out of it if he wanted.					
John's problem is a sign of personal weakness.					
John's problem is not a real medical illness.					
John is dangerous.					
It is best to avoid John so that you don't develop this problem yourself.					
John's problem makes him unpredictable.					
I would not tell anyone if I had a problem like John's.					

7. **The following questions ask how you would feel about spending time with John.** Would you be happy to...

	**Definitely not**	**Probably not**	**Not sure**	**Yes, probably**	**Yes, definitely**
Move next door to John?					
Spend an evening socializing with John?					
Make friends with John?					
Go to John's house?					
Invite John around to your house?					
Work or volunteer with John?					
Have a close relationship with John?					
Have John as a father-in-law?					

The following section concerns a hypothetical person called Paula. The description below outlines how she has been recently.

Paula is 75 years old and retired. Her husband has noticed that she has problems remembering things that happened recently but recalls things from earlier in their marriage quite well. She repeats questions which she has already asked, misplaces her things and occasionally gets confused during their conversations. Sometimes Paula and her husband quarrel as she accuses him of taking her things. She lost her way once or twice whilst driving to their son's home, and has written some cheques for the wrong amount when paying bills. When her husband points out these problems to Paula, she loses her temper. She does not think she has a problem.

8. **What, if anything, do you think is wrong with Paula**?

9. **If Paula was someone I knew and cared about, I would help her.** (Circle the number that represents your response to this statement.)

1	2	3	4	5
Strongly disagree				Strongly agree

10. **Describe all the things you would do to help Paula**.

11. **How confident do you feel in helping someone with a problem like Paula's?** (Circle the number that represents your answer.)

1<br>Not at all confident	2	3	4	5<br>Extremely confident

12. **The next few questions contain statements about Paula's problem.** Please indicate how strongly you agree or disagree with each statement.

	Strongly disagree	Disagree	Neither agree nor disagree	Agree	Strongly Agree
Paula could snap out of it if she wanted.					
Paula's problem is a sign of personal weakness.					
Paula's problem is not a real medical illness.					
Paula is dangerous.					
It is best to avoid Paula so that you don't develop this problem yourself.					
Paula's problem makes her unpredictable.					
You would not tell anyone if you had a problem like Paula's.					

13. **The following questions ask how you would feel about spending time with Paula. Would you be happy to...**

	**Definitely not**	**Probably not**	**Not sure**	**Yes, probably**	**Yes, definitely**
Move next door to Paula?					
Spend an evening socializing with Paula?					
Make friends with Paula?					
Go to Paula's house?					
Invite Paula around to your house?					
Work or volunteer with Paula?					
Have a close relationship with Paula?					
Have Paula as a mother-in-law?					

14. **The next section contains statements about health problems. Please indicate whether you agree or disagree with each statement, or if you don't know.**

Anxiety disorders and depression are less common in older people than young adults.	DISAGREE	AGREE	DON'T KNOW
Most older people with a common mental illness do not get professional help.	DISAGREE	AGREE	DON'T KNOW
If a person who is depressed does not want to seek professional help, it is important to force them to if you can.	DISAGREE	AGREE	DON'T KNOW
Recovery from anxiety disorders requires facing situations which are anxiety provoking.	DISAGREE	AGREE	DON'T KNOW
If an older person who is confused repeats questions or statements over and over, it is best to ignore them.	DISAGREE	AGREE	DON'T KNOW

14. (cont)

When interacting with a person with psychosis, it is best not to offer them choices of how you can help them because it could add to their confusion.	DISAGREE	AGREE	DON'T KNOW
A good way to help a person with a drug or alcohol problem is to let them know that you strongly disapprove of their substance use.	DISAGREE	AGREE	DON'T KNOW
It is not a good idea to ask someone if they are feeling suicidal in case you put the idea in their head.	DISAGREE	AGREE	DON'T KNOW
It is best to get someone having a panic attack to breathe into a paper bag.	DISAGREE	AGREE	DON'T KNOW
If an older person is experiencing delirium, it is helpful to reduce distracting noises, such as radio and television.	DISAGREE	AGREE	DON'T KNOW
If an older person is unsafe driving due to confusion, it is helpful to keep the person driving longer by acting as a 'co-pilot' (e.g. by giving instruction and directions to the person when they drive).	DISAGREE	AGREE	DON'T KNOW
Loud noises, an over-stimulating environment, or physical clutter may cause agitated behaviour in an older person.	DISAGREE	AGREE	DON'T KNOW
It is best not to try to reason with a person having delusions.	DISAGREE	AGREE	DON'T KNOW
If a person is intoxicated with alcohol, it is not possible to make them sober up more quickly by giving them strong coffee, a cold shower or taking them for a walk.	DISAGREE	AGREE	DON'T KNOW

15. On a scale from 'not at all new' to 'mostly new', how new was the information in the course to you?

1	2	3	4	5
Not at all new		Somewhat new		Mostly new

16. On a scale from 'none of it' to 'most of it' how much of the information in the program did you understand?

1	2	3	4	5
None of it		Some of it		Most of it

17. On a scale from ‘very poorly’ to ‘very well’ how well was the program presented?

1 Very poorly	2	3 Somewhat well	4	5 Very well

18. On a scale from ‘not very much’ to ‘very much’ how relevant was the content for you?

1 Not very much	2	3 Somewhat	4	5 Very much

19. Please rate how much you liked the following parts of the program:

	1 Not very much	2	3 Somewhat	4	5 Very much
Handbook					
Powerpoint slides					
Videos					
Activities					

20. What did you find most helpful?

21. Is there anything that could be improved?

Please take this page with you

Thank you for completing this survey!

If taking this survey brings up difficult emotions, you may wish to contact **Lifeline on 13 11 14** or **Suicide Call Back Service on 1300 659 467** for free online and telephone counselling, available 24 hours a day, seven days a week.

Please also contact the Research Manager, Alyssia Rossetto on tel: 03 9079 0207 or email: participate@mhfa.com.au so that we can keep a log of any adverse events.

Evaluating the Mental Health First Aid course for the Older Person

(Survey T3)

1. **Please provide us with your name and email address.** (We collect this data in order to match your surveys. Once your surveys are matched, your name is deleted from the information in the survey. Only the research officers have access to identifying information, we **do not** share any identifying information/data with your employers, colleagues, community members, etc.).

Name

Email

The following section concerns a hypothetical person called John. The description below outlines how he has been recently.

John is 70 years old. He has been feeling unusually sad and miserable for the last few weeks. Even though he is tired all the time, he has trouble sleeping nearly every night. John doesn't feel like eating and has lost weight. He can't keep his mind on things and puts off making decisions. Even day-to-day tasks seem too much for him. John's wife is concerned about the changes she has seen in him. John feels he will never be happy again and believes his family would be better off without him. John has been so desperate, he has been thinking of ways to end his life.

2. **What, if anything, do you think is wrong with John**?

3. **If John was someone I knew and cared about, I would help him.** (Circle the number that represents your response to this statement.)

1	2	3	4	5
Strongly disagree				Strongly agree

4. **Describe all the things you would do to help John**.

5. **How confident do you feel in helping someone with a problem like John's?** (Circle the number that represents your answer.)

1	2	3	4	5
Not at all confident				Extremely confident

6. **The next few questions contain statements about John's problem.** Please indicate how strongly you agree or disagree with each statement.

	Strongly disagree	Disagree	Neither agree nor disagree	Agree	Strongly Agree
John could snap out of it if he wanted.					
John's problem is a sign of personal weakness.					
John's problem is not a real medical illness.					
John is dangerous.					
It is best to avoid John so that you don't develop this problem yourself.					
John's problem makes him unpredictable.					
I would not tell anyone if I had a problem like John's.					

7. **The following questions ask how you would feel about spending time with John.** Would you be happy to...

	**Definitely not**	**Probably not**	**Not sure**	**Yes, probably**	**Yes, definitely**
Move next door to John?					
Spend an evening socializing with John?					
Make friends with John?					
Go to John's house?					
Invite John around to your house?					
Work or volunteer with John?					
Have a close relationship with John?					
Have John as a father-in-law?					

The following section concerns a hypothetical person called Paula. The description below outlines how she has been recently.

Paula is 75 years old and retired. Her husband has noticed that she has problems remembering things that happened recently but recalls things from earlier in their marriage quite well. She repeats questions which she has already answered, misplaces her things and occasionally gets confused during their conversations. Sometimes Paula and her husband quarrel as she accuses him of taking her things. She lost her way once or twice whilst driving to their son's home, and has written some cheques for the wrong amount when paying bills. When her husband points out these problems to Paula, she loses her temper. She does not think she has a problem.

8. **What, if anything, do you think is wrong with Paula**?

9. **If Paula was someone I knew and cared about, I would help her.** (Circle the number that represents your response to this statement.)

1<br>Strongly disagree	2	3	4	5<br>Strongly agree

10. **Describe all the things you would do to help Paula**.

11. **How confident do you feel in helping someone with a problem like Paula's?** (Circle the number that represents your answer.)

1<br>Not at all confident	2<br>A little bit confident	3<br>Moderately confident	4<br>Quite a bit	5<br>Extremely confident

12. **The next few questions contain statements about Paula's problem.** Please indicate how strongly you agree or disagree with each statement.

	Strongly disagree	Disagree	Neither agree nor disagree	Agree	Strongly Agree
Paula could snap out of it if she wanted.					
Paula's problem is a sign of personal weakness.					
Paula's problem is not a real medical illness.					
Paula is dangerous.					
It is best to avoid Paula so that you don't develop this problem yourself.					
Paula's problem makes her unpredictable.					
You would not tell anyone if you had a problem like Paula's.					

13. **The following questions ask how you would feel about spending time with Paula. Would you be happy to...**

	**Definitely not**	**Probably not**	**Not sure**	**Yes, probably**	**Yes, definitely**
Move next door to Paula?					
Spend an evening socializing with Paula?					
Make friends with Paula?					
Go to Paula's house?					
Invite Paula around to your house?					
Work or volunteer with Paula?					

13. **The following questions ask how you would feel about spending time with Paula (cont.) Would you be happy to...**

Have a close relationship with Paula?					
Have Paula as a mother-in-law?					

14. **The next section contains statements about health problems. Please indicate whether you agree or disagree with each statement, or if you don't know.**

Anxiety disorders and depression are less common in older people than young adults.	DISAGREE	AGREE	DON'T KNOW
Most older people with a common mental illness do not get professional help.	DISAGREE	AGREE	DON'T KNOW
If a person who is depressed does not want to seek professional help, it is important to force them to if you can.	DISAGREE	AGREE	DON'T KNOW
Recovery from anxiety disorders requires facing situations which are anxiety provoking.	DISAGREE	AGREE	DON'T KNOW
If an older person who is confused repeats questions or statements over and over, it is best to ignore them.	DISAGREE	AGREE	DON'T KNOW
When interacting with a person with psychosis, it is best not to offer them choices of how you can help them because it could add to their confusion.	DISAGREE	AGREE	DON'T KNOW
A good way to help a person with a drug or alcohol problem is to let them know that you strongly disapprove of their substance use.	DISAGREE	AGREE	DON'T KNOW
It is not a good idea to ask someone if they are feeling suicidal in case you put the idea in their head.	DISAGREE	AGREE	DON'T KNOW
It is best to get someone having a panic attack to breathe into a paper bag.	DISAGREE	AGREE	DON'T KNOW
If an older person is experiencing delirium, it is helpful to reduce distracting noises, such as radio and television.	DISAGREE	AGREE	DON'T KNOW

14. **The next section contains statements about health problems (cont.) Please indicate whether you agree or disagree with each statement, or if you don't know.**

If an older person is unsafe driving due to confusion, it is helpful to keep the person driving longer by acting as a 'co-pilot' (e.g. by giving instruction and directions to the person when they drive).	DISAGREE	AGREE	DON'T KNOW
Loud noises, an over-stimulating environment, or physical clutter may cause agitated behaviour in an older person.	DISAGREE	AGREE	DON'T KNOW
It is best not to try to reason with a person having delusions.	DISAGREE	AGREE	DON'T KNOW
If a person is intoxicated with alcohol, it is not possible to make them sober up more quickly by giving them strong coffee, a cold shower or taking them for a walk.	DISAGREE	AGREE	DON'T KNOW

15. **Over the last 6 months, has any older person (aged 65+) you know had any sort of mental health problem?** *(a "mental health problem" is a cluster of symptoms that affects a person's thinking, emotional state and behaviour, and disrupts the person's ability to work or carry out other daily activities and engage in satisfying personal relationships. The problem lasts for a period of weeks or more. This could include problems involving depression, anxiety, dementia, psychosis, alcohol or drug use, or suicide).*

☐ Yes (Go to question 16)

☐ No (Go to question 25)

☐ Don't know (Go to question 25)

☐ I'd rather not say (Go to question 25)

16. **Did you notice a mental health problem in just one older person or have you seen these problems in more than one person?**

☐ More than one, please specify how many _______ (Go to question 17)

☐ Just one (Go to question 18)

☐ I'd rather not say (Go to question 25)

17. **Because you know more than one older person who had a mental health problem, for the next few questions, I'd like you to THINK ABOUT THE PERSON YOU KNOW BEST**.

18. **How old is that person?** (If you don't know exactly, your best guess is fine.)

☐ 65-69 years old

☐ 70-79 years old

☐ 80 years old or over

19. **Are they**:

☐ Male

☐ Female

☐ They identify with another term

☐ Don't know

20. **Would you describe this person as:**

☐ A family member

☐ A friend

☐ A client at your work

☐ A colleague or previous colleague

☐ Other, please specify: ________________

21. **What do you think their mental health problem was?**

22. **Over the last 6 months, did you try to help the person with this problem?**

☐ Yes (Go to question 24)

☐ No (Go to question 23

23. **If you didn't help the person, are there any particular reasons that you did not try to help the person with this problem? If so, please describe these reasons.** (Go to question 25.)

24. **If you did help the person, describe all the things you did to help.**

25. At the MHFA course you attended, you received an Older Person MHFA (OMHFA) Manual. How much of the OMHFA manual did you read?

1<br>None of it	2<br>Part of it	3<br>Most of it	4<br>All of it

26. How easy was the OMHFA manual to understand?

1<br>Very difficult	2<br>Difficult	3<br>Neither difficult nor easy	4<br>Easy	5<br>Very easy

27. Did you learn much from the OMHFA manual?

1<br>Almost nothing	2<br>Not very much	3<br>A fair bit	4<br>A great deal

28. How useful was the OMHFA manual?

1<br>Not at all useful	2<br>Not very useful	3<br>Useful	4<br>Very useful

29. Do you think you will use the OMHFA manual in the future?

- ☐ No
- ☐ Yes
- ☐ Not sure

30. What have you done with the OMHFA manual? Please choose all that apply.

- ☐ Kept it
- ☐ Lent it to someone
- ☐ Given it away
- ☐ Thrown it away
- ☐ Lost it
- ☐ Don't know

31. Would you recommend the OMHFA manual to others?

1<br>Definitely not	2<br>Probably not	3<br>Probably	4<br>Yes, definitely

32. What did you like about the OMHFA manual?

33. What did you dislike about the OMHFA manual?

Please keep this page

Thank you for completing this survey!

If taking this survey brings up difficult emotions, you may wish to contact **Lifeline on 13 11 14** or **Suicide Call Back Service on 1300 659 467** for free online and telephone counselling, available 24 hours a day, seven days a week.

Please also contact the Research Officer, Kathy Bond on tel: 03 9079 0207 or email: kathybond@mhfa.com.au so that we can keep a log of any adverse events.
