# Supplementary material for: Evaluation of the Older Person Mental Health First Aid Course: Effects on Knowledge, Stigmatizing Attitudes, and Helping Behaviors
Source: J Appl Gerontol. 2025 Jul 8;45(5):856–67. doi: 10.1177/07334648251352309 (PMC13076979; doi:10.1177/07334648251352309)
Supplement: Supplemental Material - Evaluation of the Older Person Mental Health First Aid Course: Effects on Knowledge, Stigmatizing Attitudes, and Helping Behaviors [file sj-pdf-2-jag-10.1177_07334648251352309.pdf]

Scoring Criteria for Open-Ended Mental Health First Aid Responses to a Dementia Vignette

General scoring criterion: If response is to give the acronym "ALGEE", but nothing else, give 1 point per action, i.e. total of 6. Only give extra points for an action where specific detail is given.

Component of mental health first aid response	0 points (no mention or inadequate response)	1 point (superficial)	2 points (specific detail)
**A** *(approach the person)*	No mention of how to approach the person.	Mentions **any one** of the following:<br>• Considering or making an approach or engaging the person (includes gathering information before making an approach)<br>• The setting or timing (e.g. choose a suitable time or place; a familiar place; enlist help of another person)<br>• Making the approach when the person is in a suitable state (e.g. when the person is relaxed, focussed or not tired)<br>• The content of the approach (e.g. express your concerns; ask about memory; begin conversation with neutral topics; make person feel at ease).	Mentions **more than one** of the following:<br>• Considering, preparing or making an approach or engaging the person (includes gathering information before making an approach)<br>• The setting or timing (e.g. choose a suitable time or place; a familiar place; enlist help of another person)<br>• Making the approach when the person is in a suitable state (e.g. when the person is relaxed, focussed or not tired)<br>• The content of the approach (e.g. express your concerns; ask about memory; begin conversation with neutral topics; make person feel at ease).
**A** *(assess and assist with any crisis)*	Assessment/observation of risk or dealing with crises not mentioned.	Mentions assessing/observing risk or dealing with crises, but no details are given about what risks are assessed or crises dealt with.	Mentions specific risks that are assessed/observed or crises dealt with, or actions taken to deal with the crises. These could be any of the following:<br>• Unsafe behaviour due to confusion (e.g. wandering, unsafe to drive, unsafe in living situation)<br>• Challenging behaviour due to confusion (e.g. resistance, arguments, agitation, anger and aggression, disinhibited behaviour)<br>• Delirium (e.g. rapid worsening of confusion)<br>• Other crises (e.g. suicidal feelings, panic attack).
**L** *(listen and communicate non-judgementally)*	Listening to or discussing with person not mentioned, or negative type of interaction mentioned (e.g.	Mentions listening to or discussing with the person, but no details are given about the quality of the interaction, e.g. "Talk to her", "Listen to problems".<br>(Telling the person your concerns should be	Mentions listening to or discussing with the person, but also gives some specification of how this should be done or the quality of the interaction. Examples include:<br>• Listen empathically<br>• Accept the person

	"Confront them about it"), or dismissive response (e.g. "You don't need to worry about it").	scored under 'A—approach the person').	• Not judging, not being critical, or not expressing frustration<br>• Validate feelings/experience<br>• Not being confrontational<br>• Have a positive attitude<br>• Listen calmly<br>• Use neutral non-verbal skills such as open stance or comfortable eye contact<br>• Mentions specific communication techniques appropriate for a confused person (e.g. position at person's eye level, use concrete words, focus on one idea at a time, use short sentences and pauses).
**G** *(give support and information)*	Support and information not mentioned	Mentions giving support or information from **only one** of the following categories:<br>• Emotional/social support (e.g. empathizing with how they feel, acknowledge as a real problem)<br>• Practical help with tasks (e.g. accompanying to professional appointment, helping person with their memory problem, helping person compete tasks they are having problems with; helping with decisions or planning for the future)<br>• Offers resources or information (e.g. information about sources of professional help).<br><br>N.B. While *giving information* about health professionals is scored here, don't count *recommending or suggesting* professionals (as this is 'E-- encourage appropriate professional help').	Mentions giving support or information from **more than one** of the following categories:<br>• Emotional/social support (e.g. empathizing with how they feel, acknowledge as a real problem)<br>• Practical help with tasks (e.g. accompanying to professional appointment, helping person with their memory problem, helping person compete tasks they are having problems with; helping with decisions or planning for the future)<br>• Offers resources or information (e.g. information about sources of professional help).

**E** *(encourage appropriate professional help)*	Professional or getting outside help not mentioned.	Mentions professional help/getting outside help (whether "encourage", "refer", "engage" or just "help/outside help"), but does not specify type of professional help (e.g. "health professional"). Includes encouraging the family to get the person to professional help.	Mentions recommending or referring to specific type of help, or specifies "appropriate" professional help. Also includes encouraging the family to get the person to a specific type of professional help. Specific types of help are:<br>• GP/doctor/medical<br>• Psychiatrist/Psychogeriatrician<br>• Geriatrician<br>• Psychologist/ Social Worker<br>• Aged Care Assessment Team<br>• Aged care nurse<br>• Specialist.
**E** (*encourage other supports)*	Other supports not mentioned.	Mentions **only one** of the following:<br>• Support from family or friends (includes encouraging family or friends to attend a health professional to learn how to help the person; also includes providing emotional support to person in caring role)<br>• Support organizations and services (e.g. respite, day centres, supported residential care).	Mentions **both** of the following:<br>• Support from family or friends (includes encouraging family or friends to attend a health professional to learn how to help the person; also includes providing emotional support to person in caring role)<br>• Support organizations and services (e.g. respite, day centres, supported residential care).
