# Supplementary material for: Evaluation of the Older Person Mental Health First Aid Course: Effects on Knowledge, Stigmatizing Attitudes, and Helping Behaviors
Source: J Appl Gerontol. 2025 Jul 8;45(5):856–67. doi: 10.1177/07334648251352309 (PMC13076979; doi:10.1177/07334648251352309)
Supplement: Supplemental Material - Evaluation of the Older Person Mental Health First Aid Course: Effects on Knowledge, Stigmatizing Attitudes, and Helping Behaviors [file sj-pdf-3-jag-10.1177_07334648251352309.pdf]

Supplementary file 3 – course satisfaction and manual usage data

Course satisfaction:

Qualitative feedback

Table 1. Qualitative feedback on what participants thought could be improved and what they liked about the course.

Theme	N (%)	Illustrative quote
**Improvements**		
No changes	43 (54.43)	*“No, I don't think so”*
Positive feedback	19 (24.05)	*“Things could always be improved but a good course”*
Length of the course	8 (10.13)	*“Timeframe - more please!”*
Additional day	5	
More time between days	3	
Changes to content	18 (22.78)	
Adding content about specific mental health problems	5	*“A bit more focus on drugs and the affects [sic] on Mental Health, more than just alcohol when referring to substance use.”*
More activities	5	*More role play/scenarios/interactive.*
**What participants liked**		
General praise of content	31 (30.09)	*“Content was exceptional (long overdue)”*
Presentation skill of the instructor	28 (27.18)	*“Presenter [instructor name] was excellent, personable and honest in giving [their] own experiences to help relate to real life situations.”*
Discussion	22 (21.36)	*“Having real peoples stories.”*
Skills learnt	16 (15.53)	*“Learning how/what actions to take”*
Handbook /manual	15 (14.56)	*“Handbook was informative.”*
Videos	14 (13.59)	*“Love the videos, especially real life examples”*
Everything	14 (13.59)	*“I found the entire course very interesting and most helpful.”*

Note: Only responses that received endorsement by at least 10% of the sample are listed.

Quantitative feedback:

Table 2. Quantitative feedback on aspects of the course.

Question	Rating – M (SD)
How new was the information[a]	3.79 (0.96)
How much did you understand[b]	4.68 (0.70)
How well was it presented[c]	4.88 (0.39)
How much was relevant[d]	4.78 (0.46)
How well did the participants like the[d]:	
Handbook	4.90 (0.33)
PowerPoint	4.73 (0.55)
Films	4.88 (0.38)
Activities	4.67 (0.65)

Note. a: Range 1: not at all new, 5: mostly new, b: Range 1: none of it. 5: most of it, c: Range 1: very poorly, 5: very well, d: Range 1: not very much, 5: very much

Six-month follow up use of the Older Persons MHFA manual:

Qualitative feedback:

Table 3. Qualitative feedback on what participants liked and did not like about the manual.

Theme	N (%)	Illustrative quote
**Responses to what they liked:**		
Clear writing	10 (11.11)	*"Explains everything very clearly."*
Easy to read	10 (11.11)	*"It was very easy to read."*
Clear layout	11 (12.21)	*"Clear sections with actions broken out according to conditions."*
Easy to find information	12 (13.33)	*"It's set out beautifully with easy to follow guidelines if I need to quickly look up something I can find it"*
Informative	14 (15.56)	*"Informative with scenarios to help fully understand."*
**Responses to did not like:**		
Nothing to change	37 (72.55)	*"Nothing to dislike, an invaluable resource!"*

Note: Only responses that received endorsement by at least 10% of the sample are listed.

Quantitative feedback:

Table 4. How participants used the manual after the course finished.

What participants did with the manual	N (%)[ab]
Kept it	62 (92.5)
Lent it to someone	4 (5.9)
Gave it away	0
Threw it away	0
Lost it	0
Don't know	1 (1.5)

Note. a: n=65 responses from participants at six-month follow up
b: Participants were allowed to choose more than one option

Table 5: Feedback on aspects of the manual.

Question	Rating – M (SD)
How much of the manual did you read[a]	2.97 (0.68)
How much did you understand[b]	4.14 (0.66)
How much did you learn from the manual[c]	3.47 (0.59)
How useful was the manual[c]	3.45 (0.61)
Would you recommend the manual to others[d]:	3.75 (0.56)
Would you use the manual in future – yes respondents[e]	55 (87.3%)[f]

Note. a: Range 1: none of it, 4: all of it, b: Range 1: very difficult. 5: very easy, c: Range 1: almost nothing, 4: a great deal, d: Range 1: definitely not, 4: yes, definitely, e: Range: yes, no or unsure, f: number and percentage. **Based on 65 responses from six-month follow up data.**
